# Psychological distress in Nepalese residents during COVID-19 pandemic: a community level survey

**DOI:** 10.1186/s12888-020-02904-6

**Published:** 2020-10-06

**Authors:** Dhan Bahadur Shrestha, Bikash Bikram Thapa, Nagendra Katuwal, Bikal Shrestha, Chiranjibi Pant, Bina Basnet, Pankaj Mandal, Amol Gurung, Ankita Agrawal, Ramhari Rouniyar

**Affiliations:** 1 Mangalbare Hospital, Morang, Nepal; 2 Nepalese Army Institute of Health Sciences (NAIHS), Shree Birendra Hospital, Chhauni, Kathmandu, Nepal; 3 Gandhi International Mission Nepal, Kathmandu, Nepal; 4 Apex Hospital, Itahari, Nepal

**Keywords:** COVID-19, Mental health, Nepal, Pandemics psychological distress

## Abstract

**Background:**

COVID-19 pandemic has created unprecedented health and economic impact. Psychological stress, anxiety and depression are affecting not only COVID-19 patients but also health professionals, and general population. Fear of contracting COVID-19, forced restrictive social measures, and economic hardship are causing mental trauma. Nepal is a developing country from South Asia where the COVID-19 pandemic is still evolving. This online survey has been carried out to understand impact of COVID- 19 on mental health of Nepalese community dwellers.

**Methods:**

The COVID-19 Peritraumatic Distress Index (CPDI) questionnaire adapted from the Shanghai Mental Health Centre was used for online data collection from 11 April-17 May 2020. Collected data were extracted to Microsoft excel-13 and imported and analyzed using SPSS (Statistical Package for Social Sciences) version-22. An initial univariate analysis was conducted for all variables to assess the distribution. Logistic regression analyses were done to estimate the odds ratios of relevant predicting variables.

**Results:**

A total of 410 participants completed the self-rated questionnaires. Mean age of study participants was 34.8 ± 11.7 years with male preponderance. 88.5% of the respondents were not in distress (score less than 28) while, 11% had mild to moderate distress and 0.5% had severe distress. The prevalence of distress is higher among age group > 45 years, female gender, and post-secondary education group. Health professional were more likely to get distressed. Respondents with post-secondary education had higher odds (OR = 3.32; *p* = 0.020) of developing distress as compared to respondents with secondary education or lower.

**Conclusion:**

There is lower rate of psychological distress in city dwellers and people with low education. Adequate intervention and evaluation into mental health awareness, and psychosocial support focused primarily on health care workers, female and elderly individuals is necessary.

## Background

At the end of 2019, a cluster of pneumonia cases diagnosed at Wuhan rapidly turned into epidemic in China. World Health Organization named this disease- ‘COVID-19’ (Corona Virus Disease- 2019) and the novel virus- severe acute respiratory syndrome coronovisurs-2 (SARS-cov-2) [1]. The outbreak was declared a ‘Public Health Emergency of International Concern’ on 30 January 2020. On March 11 ‘COVID-19 Pandemic’ was declared when approximately 118,000 cases were reported in more than 110 countries and territories [2]. The spectrum of symptomatic infection ranges from mild to severe. The epidemiology has heterogeneous socio-economic distribution and clinical presentation. Most infections are not severe. Nepal registered the index case of COVID-19 on January 23, 2020 and first mortality on May 17, 2020. After the isolation of the second case on 23 March, Nepal underwent strict restrictive measures like nationwide lockdown, social distancing, and travel restriction. Till May 27, 2020 Nepal registered 772 COVID-19 confirmed cases with four COVID-19 related mortality. The case fatality rate was 0.5% and recovery rate was 17.9% [3, 4]. In a study conducted in Nepal during lockdown of COVID-19 pandemic; depression, anxiety and depression and anxiety co-morbidity was reported to be 34.1, 31.2 and 23.2% respectively. Health professionals had 1.7 times, 2 times and 3.4 times higher odds of depression, anxiety and depression and depression-anxiety co-morbidity respectively compared to others [5].

The COVID-19 pandemic has forced people in social distancing and isolation; health and economic crisis; and ‘infodemics’, irrespective of profession, origin, and religion. Patients, health professionals, and the general public are under unprecedented mental pressure that may result into spectrum of short and long term psychological health issues like anxiety, stress, depression, panic attack, and post-traumatic stress disorder [6, 7]. This study has been conducted to find out the impact of COVID-19 on mental health of residents of Nepal during lockdown.

## Methods

This is nationwide survey of psychological distress in the general population of Nepal during the COVID-19 pandemic. The COVID-19 Peritraumatic Distress Index (CPDI) questionnaire (survey questionnaire attached as Supplementary file) adapted from the Shanghai Mental Health Centre [8]. The CPDI questionnaire incorporated relevant diagnostic guidelines for specific phobias and stress disorders specified in the International Classification of Diseases, 11th Revision. The survey data is collected through online Google Form with informed consent. The structured Google Form with CPDI question is published in social media network and sent in personal mail requesting participants to share the survey form to wider audiences. The psychological distress score is made available to respondents upon completion of the questionnaire. The Google form was used to collect demographic data (age, gender, religion, education, employment status, monthly family income, nationality, ethnicity, and residence), and the response to CPDI questionnaire. The CPDI questionnaire registered details including anxiety, depression, specific phobias, cognitive change, avoidance, and compulsive behaviour, physical symptoms and loss of social functioning in the past week. The overall response was indexed and categorized in three different group (mild, moderate, severe) of psychological distress level with score ranging from 0 to 96. A score ≤ 28 is normal; 29 to 51 is mild to moderate distress, and ≥ 52 is severe distress. Psychiatrists and public health physicians from Nepal verified the content validity of the CPDI. The linguistic validation of the questionnaire was done with forward translation by two independent translators, reconciliation, and again backward translated by two independent translators who are blind to the original questionnaire format. The approved translation was put alongside the original language questionnaire. Data were collected from 11 April (3 weeks since enforcement of nationwide lockdown) till 17 May 2020.

### Exposure variable

The survey questionnaire included socio-economic and demographic variables such as age (< 30, 30–45, > 45), gender (male, female), religion (Hinduism and non-Hinduism), education (less than secondary, post-secondary and tertiary education), employment status (employed, not-employed, students), monthly family income (<NRS100900, NRS 100901-309000and > NRS 309000), nationality (Nepali and non-Nepali), ethnicity (Brahmin & Chettri and Other), and residence (Province1; 2; 3;4;5;6;&7)

### Outcome variable

Modified version of the COVID-19 Peri-traumatic Distress Index (CPDI) with 24 items is used to measure outcome. The present study has used the e-questionnaire in Nepali version (supplemented with English version) of the CPDI, and internal consistency was assessed by using Cronbach’s α. The internal reliability of the present study found to be 0.896 (*p* < 0.001). For each of the 24 items, participants were asked to self-rate psychological impact related to COVID-19 and frequency activities in the last week. The 5-point Likert scoring system was used (never-0, occasionally-1, sometimes-2, often-3, always-4) to rate the psychological impact. A score of 0–28 is normal or no distress. A total score between 29 and 51 indicates mild to moderate distress and a score of greater than and equal to 52 indicates severe distress.

### Statistical analysis

Collected data were extracted to Microsoft excel-13 and imported and analysed using SPSS (Statistical Package for Social Sciences) version-22). An initial univariate analysis was conducted for all variables to assess the distribution for each variable. Categorical variables were summarized using percentages. Logistic regression analyses were used to estimate the odds ratios of relevant predicting variables. This gives how a set predictor X (exposure variables) is related to the dichotomous response variable of Y (outcome variable). For convenience, we define the response to be Y = 0 or 1, with Y = 1 denoting the occurrence of the event of interest. The outcome variable is No distress = 0 and distress = 1. The exposure variable were continuous as well as categorical.

### Research ethics

All respondents gave their informed consent for inclusion before they participated in the study. The study was conducted in accordance with the protocol and approved by the Ethics Committee of Nepalese Army Institute of Health Sciences (NAIHS).

## Results

A total of 410 participants completed all the self-rated questionnaires. Table 1 present the socio-demographic profile of respondents who participated in the study. In our study, mean age of study participants was 34.8 *±* 11.7 years ranging from 17 to 83 years. The majority of the participants were male (64.6%; *n* = 265). The 79% of the respondents were educated (post-secondary and higher) (*n* = 324), and 90% were Hindu by religion. Among study participants 70% were employed and 40.7% were health care workers. About two third of respondent were resident of Bagmati Province (*n* = 258, 62.9%) (Table 1).

**Table 1:** Socio-demographic profile of the respondents (*N* = 410)

Socio-demographic Variables	n	%
Age (in Years)		
< 30	163	39.8
30–45	188	45.9
> 45	59	14.4
*Mean ± SD*	34.8 *±* 11.7	
Gender		
Female	144	35.1
Male	265	64.6
Other	1	0.2
Religion		
Hinduism	369	90.0
Non-Hinduism	41	10.0
Education		
*Less than secondary*	86	21.0
*Post-secondary education*	131	32.0
*Tertiary education*	193	47.1
Employment status		
Employment	290	70.7
Non-employment	30	7.3
Student	90	22.0
Monthly family income		
< NRS 100900	374	91.2
> NRS 100900–309,000	21	5.1
*>* NRS 309000	15	3.7
Nationality		
Nepali	403	98.3
Non-Nepali	7	1.7
Ethnicity		
Bhramin and Chettri	252	61.5
Other	158	38.5
Residence		
Province 1 (Briatnagar as territorial capital)	67	16.3
Province 2 (Janakpur as territorial capital)	37	9.0
Province 3 (Bagmati)	258	62.9
Province 4 (Gandaki)	21	5.1
Province 5 (Butwal as territorial capital)	13	3.2
Province 6 (Karnali)	4	1.0
Province 7 (Sudurpaschim)	10	2.4
Are you a healthcare worker?		
Yes	167	40.7
No	243	59.3
**Total**	**410**	**100**

NB: Nepal is yet to name all the provinces under the mandate of new constitution and federal People’s Republic

Table 2 depicts the prevalence of each psychological components of CPDI. Fifty percent (*n* = 205) were nervous, anxious at some moments and bought a lot of masks, medications, sanitizers, gloves, and other home supplies during the COVID-19 pandemic crisis. Two third (*n* = 271, 66.1%) of study population were worried about their families being infected and continue updating with COVID-19 related news and information (60.4%). Majority (*n* = 361, 88%), of respondents felt sympathetic to COVID-19 patients and their families.

**Table 2:** Presence of symptoms COVID- 19 Peri-traumatic distress (CPDI)

	Never n(%)	Occasionally n(%)	Sometimes n(%)	Often n(%)	Always n(%)
Question 1: Compared to usual, I feel more nervous and anxious.	200(48.8)	166(40.5)	30(7.3)	13(3.2)	1(0.2)
Question 2: I feel insecure and bought a lot of masks, medications, sanitizers, gloves and/or other home supplies.	205(50.0)	122(29.8)	64(15.6)	8(2.0)	11(2.7)
Question 3: I can’t stop myself from imagining myself or my family being infected and feel terrified and anxious about it.	139(33.9)	177(43.2)	75(18.3)	14(3.4)	5(1.2)
Question 4: I feel helpless no matter what I do.	291(71.0)	82(20.0)	27(6.6)	9(2.2)	1(0.2)
Question 5: I feel sympathetic to COVID-19 patients and their families.	49(12.0)	81(19.8)	99(24.1)	58(14.1)	123(30.0)
Question 6: I feel helpless and angry about people around me, governors, and media.	133(32.4)	153(37.3)	75(18.3)	39(9.5)	10(2.4)
Question 7: I am losing faith in the people around me.	213(52.0)	113(27.6)	65(15.9)	16(3.9)	3(0.7)
Question 8: I collect information about COVID-19 all day. Even if it’s not necessary, I can’t stop myself.	162(39.5)	127(31.0)	68(16.6)	29(7.1)	24(5.9)
Question 9: I will believe the COVID-19 information from all sources without any evaluation.	284(69.3)	85(20.7)	32(7.8)	9(2.2)	0(0.0)
Question 10: I would rather believe in negative news about COVID-19 and be skeptical about the good news.	310(75.6)	63(15.4)	28(6.8)	7(1.7)	2(0.5)
Question 11: I am constantly sharing news about COVID-19 (mostly negative news).	276(67.3)	80(19.5)	36(8.8)	11(2.7)	7(1.7)
Question 12: I avoid watching COVID-19 news since I am too scared to do so.	281(68.5)	82(20.0)	34(8.3)	12(2.9)	1(0.2)
Question 13: I am more irritable and have frequent conflicts with my family.	294(71.7)	79(19.3)	23(5.6)	11(2.7)	3(0.7)
Question 14: I feel tired and sometimes even exhausted.	227(55.4)	125(30.5)	37(9.0)	19(4.6)	2(0.5)
Question 15: When feelings anxious, my reactions are becoming sluggish.	277(67.6)	97(23.7)	24(5.9)	11(2.7)	1(0.2)
Question 16: I find it hard to concentrate.	233(56.8)	121(29.5)	31(7.6)	21(5.1)	4(1.0)
Question 17: I find it hard to make any decisions.	277(67.6)	92(22.4)	31(7.6)	9(2.2)	1(0.2)
Question 18: During this COVID-19 period, I often feel dizzy or have back pain and chest distress.	317(77.3)	64(15.6)	22(5.4)	7(1.7)	0(0.0)
Question 19: During this COVID-19 period, I often feel stomach pain, bloating, and other stomach discomforts.	299(72.9)	81(19.8)	21(5.1)	8(2.0)	1(0.2)
Question 20: I feel uncomfortable when communicating with others.	311(75.9)	66(16.1)	25(6.1)	7(1.7)	1(0.2)
Question 21: Recently, I rarely talk to my family.	325(79.3)	57(13.9)	17(4.1)	9(2.2)	2(0.5)
Question 22: I have frequent awakening at night due to my dream about myself or my family being infected by COVID-19.	321(78.3)	68(16.6)	18(4.4)	2(0.5)	1(0.2)
Question 23: I have changes in my eating habits	302(73.7)	62(15.1)	31(7.6)	13(3.2)	2(0.5)
Question 24: I have constipation or frequent urination.	338(82.4)	48(11.7)	19(4.6)	3(0.7)	2(0.5)

More than two third (*n* = 277, 67.5%) of the participants felt helpless and angry about the people around them. Approximately one third of participants endorsed COVID-19 information from all sources without validation. Approximately a quarter of the respondents complained of somatic problems like dizziness, back pain, and chest distress; and felt change in their eating habits during COVID-19 period. More than two third of the respondents prefer not share negative news related to COVID-19 and not skeptical about news.

Table 3 demonstrates distribution of level of distress by socioeconomic and demographic characteristics of Nepal. The prevalence of mild to severe distress in the age group < 30 years, 30–45 years and > 45 years old were 12.2, 9.5, and 15.3% respectively. The prevalence of mild to moderate and severe distress was higher among females than males.

**Table 3:** Prevalence of CPDI by socioeconomic and demographic characteristics, Nepal

	Normal n (%)	Mild to moderate distress n (%)	Severe distress n (%)
Age			
< 30	143(87.7)	19(11.7)	1(0. 6)
30–45	170(90.4)	17(9.0)	1(0.5)
> 45	50(84.7)	9(15.3)	0(0.0)
Gender			
Female	125(86.8)	18(12.5)	1(0.7)
Male	237(89.4)	27(10.2)	1(0.4)
Other	1(100.0)	0(0.0)	0(0.0)
Religion			
Hinduism	327(88.6)	41(11.1)	1(0.3)
Non-Hinduism	36(87.8)	4(9.8)	1(2.4)
Education			
Less than Secondary	80(93.0)	6(7.0)	0(0.0)
Post-secondary	104(79.4)	27(20.6)	0(0.0)
Tertiary	179(92.7)	12(6.2)	2(1.0)
Employment			
Employment	258(89.0)	31(10.7)	1(0.3)
Non-employment	29(96.7)	1(3.3)	0(0.0)
Student	76(84.4)	13(14.4)	1(1.1)
Household’s Monthly Income			
< NRS 100900	331(88.5)	42(11.2)	1(0.3)
*>* NRS 100901–309,000	19(90.5)	2(9.5)	0(0.0)
*>* NRS 309000	13(86.7)	1(6.7)	1(6.7)
Nationality			
Nepali	359(89.1)	43(10.7)	1(0.2)
Non-Nepali	4(57.1)	2(28.6)	1(14.3)
Ethnicity			
Bhramin and Chettri	217(86.1)	33(13.1)	2(0.8)
Other	146(92.4)	12(7.6)	0(0.0)
Your State of Residence			
Province 1	50(74.6)	16(23.9)	1(1.5)
Province 2	29(78.4)	8(21.6)	0(0.0)
Province 3	241(93.4)	16(6.2)	1(0.4)
Province 4	21(100.0)	0(0.0)	0(0.0)
Province 5	11(84.6)	2(15.4)	0(0.0)
Province 6	3(75.0)	1(25.0)	0(0.0)
Province 7	8(80.0)	2(20.0)	0(0.0)
Are you a healthcare worker?			
No	211(86.8)	32(13.2)	0(0.0)
Yes	152(91.0)	13(7.8)	2(1.2)

Further, the level of distress was higher among respondents who have post-secondary education or more, than lower education group. Similarly, the prevalence of distress was higher among students (15.6%, *n* = 14), who have household income of < NRS 100900 (11.5%, *n* = 43), Brahmin and Chettri (13.9%, *n* = 35), non-Nepali (42.9%, n = 3), and resident of province one (25.4%, *n* = 17).

**Fig. 1:**
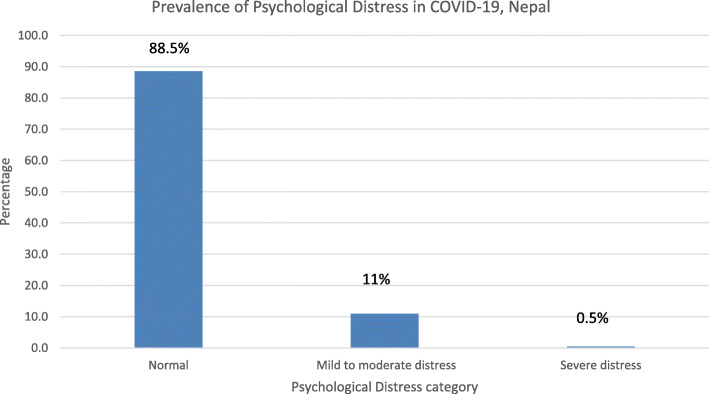
Prevalence of Psychological Distress in COVID-19, Nepal

Overall 88.5% (*n* = 363) of the participants were not distressed while, 11% (*n* = 45) were mild to moderate distressed and 0.5% (n = 2) were severely distressed due to COVID-19 pandemic (Fig. 1).

Table 4 illustrates the predictor of distress using binary logistic regression. After adjusting for other factors, the participants residing at Bagmati province were less distressed (OR = 0.244, 95% CI: 0.111–0.539, *p* = 0.000) compared to participants residing at province one.

**Table 4:** Predictors of CPDI through binary logistics regression

	Exp(B)	95% C.I. for EXP(B)		*p*-value
		Lower	Upper	
Age (in Years)				
< 30^a^				.562
30–45	1.157	.456	2.932	.759
> 45	1.819	.580	5.697	.305
Gender				
Male^a^				.876
Female	1.212	.583	2.523	.606
Other	.000	0.000		1.000
Religion				
Hinduism^a^				
Non-Hinduism	.792	.265	2.365	.677
Education				
*Less than secondary*^a^				.007
*Post-secondary education*	3.320^b^	1.208	9.124	.020
*Tertiary education*	1.152	.358	3.710	.813
Employment status				
Employment^a^				.300
Non-employment	1.283	.496	3.322	.607
Student	.237	.028	1.977	.183
Monthly family income				
< NRS 100900^a^				.865
> NRS 100900–309,000	1.043	.200	5.443	.960
*>* NRS 309000	1.654	.264	10.350	.591
Nationality				
Nepali^a^				
Non-Nepali	3.672	.667	20.235	.135
Ethnicity				
Bhramin and Chettri^a^				
Other	.510	.256	1.014	.055
Your State of Residence				
Province 1^a^				.018
Province 2	.882	.311	2.502	.814
Province 3	.244^b^	.111	.539	.000
Province 4	.000	0.000		.998
Province 5	.775	.139	4.324	.772
Province 6	1.130	.094	13.620	.923
Province 7	.842	.139	5.098	.851
Are you a healthcare worker?				
Yes^a^				
No	1.120	.515	2.434	.775
Constant	.123			.010

^a^ Reference Category, ^b^Significant at 5% level of significance

Participants with post-secondary education had 3.32 (95% CI: 1.208–9.124, *p* = 0.020) times higher risk of developing distress as compared to secondary education or lower. Other variables did not show relation to develop distress of COVID- 19 after adjusting other factors.

## Discussion

Female participants, single living, and health professionals have higher risk of developing psychological impact during the COVID-19 pandemic [5]. Study in China showed significant differences in psychological distress among different population demographics and epidemiological characteristics of disease [9]. The rate of psychological stress range from 8 to 28% with female preponderance [10]. Sociocultural inequity and gender norms; lack of access to health and education; and restricted control over economy make female more vulnerable to mental health problems in most of the low and middle income countries [11]. Eleven percent of our study participants had mild psychological distress during COVID-19 pandemic that is comparable (13.6%) to the cross-sectional study done in Liaoning Province of China [12]. ‘Theory of behavioural immune system’ explains the negative emotion and distress during COVID-19 pandemic. The risk of contracting disease, forced restrictive measures (quarantine, lockdown, social distancing restrictive measures), and economic recession are causing gradual rise in detrimental psychological effect in population [13, 14]. The varied spatio-temporal distribution of the pandemic might explain the heterogeneity in the rate of the psychological distress among different geographical region of the countries.

A high prevalence of psychological distress is noted in health professional from developing as well as developed countries [5, 15]. A poorly governed healthcare system can incite more detrimental mental and physical health impact among frontline line health professionals [16]. Scarcity of the hospital resources, long duty hours, lack of reciprocity, and compromised family care are important predictors of psychological distress in health care professionals [17]. Lack of accountable leadership and transparent communication are lowering effectiveness and efficiency of the health care system in Nepal.

The economically and physically active population group (education- post secondary or higher, students) have higher rate of psychological stress. People from province one, bordering India had higher (25.4%) percentage of mild to severe stress than other provinces. Cities from national capital region, show less risk of psychological distress (*p* < 0.02). The Education level and income level have inverse relation with level of psychological stress of the Nepalese community. Higher prevalence of distress is noted among the high impact countries [18, 19]. A meta-analysis reveal higher level of either acute or post-traumatic stress (OR = 1.71) and psychological distress (OR = 1.74) in the healthcare staff exposed to the infection [20]. Bohlken and colleagues mention that the severity of psychological symptoms depends on; individual’s age, gender, occupation, and proximity to the infected patient [21]. Older people, city dwellers, people with stable income, and living with parents present with low anxiety and depression [22, 23]. Educated young people are more likely to follow pandemic related news and information (“infodemics”) that aggravates the anxiety and panic attacks [24]. Citizens living in close contact with COVID-19 cases tend to have higher odd (OR = 3.007) of anxiety [22].

Identification of the vulnerable group and appropriate public health measure is necessary to mitigate the long term psychological effects [24, 25]. The female gender, low economy, rural community, and healthcare workers are more vulnerable in Nepal. Health education and information on epidemiology, prevention and control measures, and mental health counselling protect the public health [26]. In a span of two and half months of lockdown (23 March) a total of 1277 committed suicide in Nepal, which in average is 20% higher than the previous year [27]. The COVID-19 pandemic should be addressed with viable health, economy and social safety nets targeting vulnerable groups. The disease outbreak, media, government action, and public response, all have consequences on psychological health of population. These stakeholders need to act with responsibility. The community mental health support should be a part of the COVID-19 preparedness and response plan [27]. The comprehensive healthcare approach to COVID-19 pandemic includes: “Protection of vulnerable people; Provision of treatment and support services to affected people; Continuity of regular healthcare services for the whole population; Protection and support of primary healthcare workers and primary care services; Provision of mental health services to the community and the primary healthcare workforce” [28].

### Limitation

A validated self-reported questionnaire was used to assess the severity of the distress along with the socio-economic predictors of psychological distress in the context of early period of COVID- 19 pandemic in Nepal. The availability of internet facility, education level, and responder’s compliance might have influenced the number of participants in this study. The study sample lack representation of all geographical and economic status of Nepal. Most of the predictors of the psychological distress were not statistically significant. We did not measure the different sub-scales of CPDI in our study. The trend of pandemic inside the country is still emerging. The spatio-temporal distribution of the pandemic inside the country and its long term effect is beyond the scope of this peri-traumatic stress index. The future psychological evaluation among Nepalese population necessitates sub-categorical analysis of psychological stress with wider participants.

## Conclusion

We have found significantly lower rate of psychological distress in city dwellers and in people with low education level. The rate of psychological distress is low (11.5%) as the pandemic is still emerging. Focus and customized approach to determinants of psychological health like education and awareness, psychosocial support, self- empowerment, and professional services can break the chain of emerging psychological distress pandemic. Our study concludes that integrating public mental health services into national public health preparedness and emergency response plan, with extra focus on vulnerable groups like health care workers, female, marginalised, and older age co-morbid individuals.

## Supplementary information

**Additional file 1.** Online questionnaire.

## Data Availability

The datasets analyzed during the current study are available from the corresponding author on reasonable request.

## Abbreviations

CI: Confidence Interval
COVID-19: Coronavirus disease-19
CPDI: COVID-19 Peritraumatic Distress Index
n: Number of patients
NAIHS: Nepalese Army Institute of Health Sciences
NRS: Nepalese Rupees
OR: Odds ratio
SARS-CoV-2: Severe acute respiratory syndrome coronavirus-2
SPSS: Statistical Package for Social Sciences

## References

[1] He F, Deng Y, Li W. Coronavirus disease 2019: what we know? J Med Virol. 2020. 10.1002/jmv.25766. 10.1002/jmv.25766 PMC7228340 32170865

[2] Jin Y, Yang H, Ji W, Wu W, Chen S, Zhang W, et al. Virology, epidemiology, pathogenesis, and control of COVID-19. Viruses. 2020;12(4). 10.3390/v12040372. 10.3390/v12040372 PMC7232198 32230900

[3] Coronavirus disease (COVID-19) outbreak updates & resource materials [database on the Internet]. Health Emergecny operation centre. 2020. Available from: https://heoc.mohp.gov.np/update-on-novel-corona-virus-covid-19/.

[4] Coronavirus disease (COVID-2019) situation reports [database on the Internet] 2020. Available from: https://www.who.int/emergencies/diseases/novel-coronavirus-2019/situation-reports.

[5] Sigdel A, Bista A, Bhattarai N, Poon BC, Giri G, Marqusee H, et al. Depression, anxiety and depression-anxiety comorbidity amid COVID-19 pandemic: an online survey conducted during lockdown in Nepal. medRxiv. 2020:2020.04.30.20086926. 10.1101/2020.04.30.20086926.

[6] Galea S, Merchant RM, Lurie N. The mental health consequences of COVID-19 and physical distancing: the need for prevention and early intervention. JAMA Intern Med. 2020. 10.1001/jamainternmed.2020.1562. 10.1001/jamainternmed.2020.1562 32275292

[7] Rajkumar RP (2020). COVID-19 and mental health: a review of the existing literature. Asian J Psychiatr.

[8] Qiu J, Shen B, Zhao M, Wang Z, Xie B, Xu Y (2020). A nationwide survey of psychological distress among Chinese people in the COVID-19 epidemic: implications and policy recommendations. Gen Psychiatr.

[9] Wang H, Xia Q, Xiong Z, Li Z, Xiang W, Yuan Y (2020). The psychological distress and coping styles in the early stages of the 2019 coronavirus disease (COVID-19) epidemic in the general mainland Chinese population: a web-based survey. PLoS One.

[10] Wang C, Pan R, Wan X, Tan Y, Xu L, McIntyre RS, et al. A longitudinal study on the mental health of general population during the COVID-19 epidemic in China. Brain Behav Immun. 2020. 10.1016/j.bbi.2020.04.028. 10.1016/j.bbi.2020.04.028 PMC7153528 32298802

[11] Bradshaw S, Fordham M (2013). Women, girls and disasters - a review for DFID.

[12] Zhang Y, Ma ZF. Impact of the COVID-19 pandemic on mental health and quality of life among local residents in Liaoning Province, China: a cross-sectional study. Int J Environ Res Public Health. 2020;17(7). 10.3390/ijerph17072381. 10.3390/ijerph17072381 PMC7177660 32244498

[13] Mortensen CR, Becker DV, Ackerman JM, Neuberg SL, Kenrick DT (2010). Infection breeds reticence: The effects of disease salience on self-perceptions of personality and behavioural avoidance tendencies. Psychol Sci.

[14] Derivois D, Cénat JM, Joseph NE, Karray A, Chahraoui K (2017). Prevalence and determinants of post-traumatic stress disorder, anxiety and depression symptoms in street children survivors of the 2010 earthquake in Haiti, four years after. Child Abuse Negl.

[15] Shacham M, Hamama-Raz Y, Kolerman R, Mijiritsky O, Ben-Ezra M, Mijiritsky E. COVID-19 factors and psychological factors associated with elevated psychological distress among dentists and dental hygienists in Israel. Int J Environ Res Public Health, Doi. 2020;17(8). 10.3390/ijerph17082900. 10.3390/ijerph17145074 PMC7399825 32674416

[16] Amerio A, Bianchi D, Santi F, Costantini L, Odone A, Signorelli C (2020). Covid-19 pandemic impact on mental health: a web-based cross-sectional survey on a sample of Italian general practitioners. Acta Biomed.

[17] Anesi GL, Lynch Y, Evans L (2020). A conceptual and adaptable approach to hospital preparedness for acute surge events due to emerging infectious diseases. Crit Care Explor.

[18] Kang L, Ma S, Chen M, Yang J, Wang Y, Li R, et al. Impact on mental health and perceptions of psychological care among medical and nursing staff in Wuhan during the 2019 novel coronavirus disease outbreak: a cross-sectional study. Brain Behav Immun. 2020. 10.1016/j.bbi.2020.03.028. 10.1016/j.bbi.2020.03.028 PMC7118532 32240764

[19] Chew NWS, Lee GKH, Tan BYQ, Jing M, Goh Y, Ngiam NJH, et al. A multinational, multicentre study on the psychological outcomes and associated physical symptoms amongst healthcare workers during COVID-19 outbreak. Brain Behav Immun. 2020. 10.1016/j.bbi.2020.04.049. 10.1016/j.bbi.2020.04.049 PMC7172854 32330593

[20] Kisely S, Warren N, McMahon L, Dalais C, Henry I, Siskind D (2020). Occurrence, prevention, and management of the psychological effects of emerging virus outbreaks on healthcare workers: rapid review and meta-analysis. BMJ.

[21] Bohlken J, Schomig F, Lemke MR, Pumberger M, Riedel-Heller SG (2020). COVID-19 pandemic: stress experience of healthcare workers - a short current review. Psychiatr Prax.

[22] Cao W, Fang Z, Hou G, Han M, Xu X, Dong J (2020). The psychological impact of the COVID-19 epidemic on college students in China. Psychiatry Res.

[23] Gonzalez-Sanguino C, Ausin B, Castellanos MA, Saiz J, Lopez-Gomez A, Ugidos C, et al. Mental health consequences during the initial stage of the 2020 coronavirus pandemic (COVID-19) in Spain. Brain Behav Immun. 2020, 2020. 10.1016/j.bbi.2020.05.040. 10.1016/j.bbi.2020.05.040 PMC7219372 32405150

[24] Ahmad AR, Murad HR (2020). The impact of social media on panic during the COVID-19 pandemic in Iraqi Kurdistan: online questionnaire study. J Med Internet Res.

[25] Huang Y, Zhao N. Mental health burden for the public affected by the COVID-19 outbreak in China: who will be the high-risk group? Psychol Health Med. 2020:1–12. 10.1080/13548506.2020.1754438. 10.1080/13548506.2020.1754438 32286091

[26] Wang C, Pan R, Wan X, Tan Y, Xu L, Ho CS, et al. Immediate psychological responses and associated factors during the initial stage of the 2019 coronavirus disease (COVID-19) epidemic among the general population in China. Int J Environ Res Public Health. 2020;17(5). 10.3390/ijerph17051729. 10.3390/ijerph17051729 PMC7084952 32155789

[27] Poudel K, Subedi P (2020). Impact of COVID-19 pandemic on socioeconomic and mental health aspects in Nepal. Int J Soc Psychiatry.

[28] Kidd MR (2020). Five principles for pandemic preparedness: lessons from the Australian COVID-19 primary care response. Br J Gen Pract.

