# Supplementary material for: Psychological distress in Nepalese residents during COVID-19 pandemic: a community level survey
Source: BMC Psychiatry. 2020 Oct 6;20:491. doi: 10.1186/s12888-020-02904-6 (PMC7538049; doi:10.1186/s12888-020-02904-6)
Supplement: Supplementary file 1 — Additional file 1. Online questionnaire. [file 12888_2020_2904_MOESM1_ESM.docx]

PSYCHOLOGICAL DISTRESS DURING COVID-19 PANDEMIC AMONG NEPALESE RESIDENTS

[INVITATION TO PARTICIPATE IN AN ONLINE SURVEY TO ASSESS PSYCHOLOGICAL DISTRESS DURING COVID-19 PANDEMIC AMONG NEPAL RESIDENTS]

Introduction of Research

COVID-19 pandemic has caused serious threats to people’s physical health and lives. It may also trigger a wide range of psychological responses such as panic, anxiety, and depression. It is important to know that these responses are normal in situations like a pandemic. However, if you are experiencing worry, fear, sadness, hopelessness and other similar feelings a lot of the time for more than two weeks, it may indicate psychological distress. This study is the first nationwide large-scale survey of psychological distress in the general population of Nepal during the COVID-19 pandemic.

Purpose of Research

The main purpose of this study is to measure the prevalence and severity of this psychological distress, gauge the current mental health burden on society, and therefore provide a concrete basis for tailoring and implementing relevant mental health intervention policies to cope with this challenge efficiently and effectively.

Participation in Research, Benefits, and Risk.

Your participation in this research is entirely voluntary. You may refuse to take part in the study or you may withdraw yourself from participation in the research at any time without penalty. The outcome of the study will help to establish a crisis prevention and intervention system to reduce psychological distress and prevent further mental health problems during a pandemic. Information obtained from this research will benefit the individuals, researchers, institution and community for the advancement of knowledge and future practice. Engagement in this study is minimal risk if you follow the instruction given to you. If you would like to know the results of the study, we will notify you. However, if you have urgent problems please consult a qualified medical professional.

How long will it takes:

The procedure involves filling an online survey that will take approximately 6-8 minutes.

Confidentiality

Your information will be kept confidential by the investigators and will not be made public unless disclosure is required by law. By signing this consent form, you will authorize the review of records, analysis and use of the data arising from this research.

The research was approved by the Ethics Committee of Nepalese Army Institute of Health Sciences (NAIHS).

If you have any questions about the research or your right, please contact any following researchers:

Payment and compensation

You do not have to pay for participating in this study. Similarly, no payment is available to you for participating in this study.

Agreement to Participate

Your participation is completely voluntary, and you can withdraw at any time. To take this survey, you must be at least 18 years old and above.

If you meet the above criteria and would like to take the survey, click the button "Agree" below to start the survey

* Required

Email address *

By clicking on "Agree" I herewith confirm that: 1. I voluntarily agree to take part in this research and to provide all necessary information to the investigators as requested. 2. I may at any time choose to withdraw from this research without giving any reason. 3. I agree to hold them harmless from any harm or loss that may be incurred by me due to my participation in the research. * *

**Agree**

Name:

Date today*: MM/DD/YYYY

Thank you for agreeing to participate in this study

Your State of Residence *:

1/2/3/4/5/6/7

Nationality *:

Nepali /No-Nepali

Gender *: Male /Female/ other

Ethnicity *:

Religion *:

Age in years *:

Education *:

Primary/Secondary/Post-secondary education (pre-university/Diploma)/ Tertiary education (Degree/ Master)

Employment *:

Monthly Income *

NRS < 14700/ 14701 – 57400/ 57401 – 100900/100901- 300900/ >300901

Are you a healthcare worker? *: yes/ no

**Questions 1-24 asking your feeling on current pandemic COVID 19. Please select the most appropriate answer from the options below.**

Question 1: Compared to usual, I feel more nervous and anxious.

Best option

1. Never

2. Occasionally

3. Sometimes

4. Often

5. Always

Question 2: I feel insecure and bought a lot of masks, medications, sanitizers, gloves and/or other home supplies.

1. Never

2. Occasionally

3. Sometimes

4. Often

5. Always

Question 3: I can’t stop myself from imagining myself or my family being infected and feel terrified and anxious about it.

1. Never

2. Occasionally

3. Sometimes

4. Often

5. Always

Question 4: I feel helpless no matter what I do.

1. Never

2. Occasionally

3. Sometimes

4. Often

5. Always

Question 5: I feel sympathetic to COVID-19 patients and their families.

1. Never

2. Occasionally

3. Sometimes

4. Often

5. Always

Question 6: I feel helpless and angry about people around me, governors, and media.

1. Never

2. Occasionally

3. Sometimes

4. Often

5. Always

Question 7: I am losing faith in the people around me.

1. Never

2. Occasionally

3. Sometimes

4. Often

5. Always

Question 8: I collect information about COVID-19 all day. Even if it’s not necessary, I can’t stop myself.

1. Never

2. Occasionally

3. Sometimes

4. Often

5. Always

Question 9: I will believe the COVID-19 information from all sources without any evaluation.

1. Never

2. Occasionally

3. Sometimes

4. Often

5. Always

Question 10: I would rather believe in negative news about COVID-19 and be skeptical about the good news.

1. Never

2. Occasionally

3. Sometimes

4. Often

5. Always

Question 11: I am constantly sharing news about COVID-19 (mostly negative news).

1. Never

2. Occasionally

3. Sometimes

4. Often

5. Always

Question 12: I avoid watching COVID-19 news since I am too scared to do so.

1. Never

2. Occasionally

3. Sometimes

4. Often

5. Always

Question 13: I am more irritable and have frequent conflicts with my family.

1. Never

2. Occasionally

3. Sometimes

4. Often

5. Always

Question 14: I feel tired and sometimes even exhausted.

1. Never

2. Occasionally

3. Sometimes

4. Often

5. Always

Question 15: When feelings anxious, my reactions are becoming sluggish.

1. Never

2. Occasionally

3. Sometimes

4. Often

5. Always

Question 16: I find it hard to concentrate.

1. Never

2. Occasionally

3. Sometimes

4. Often

5. Always

Question 17: I find it hard to make any decisions.

1. Never

2. Occasionally

3. Sometimes

4. Often

5. Always

Question 18: During this COVID-19 period, I often feel dizzy or have back pain and chest distress.

1. Never

2. Occasionally

3. Sometimes

4. Often

5. Always

Question 19: During this COVID-19 period, I often feel stomach pain, bloating, and other stomach discomforts.

1. Never

2. Occasionally

3. Sometimes

4. Often

5. Always

Question 20: I feel uncomfortable when communicating with others.

1. Never

2. Occasionally

3. Sometimes

4. Often

5. Always

Question 21 - I talked with my family members very rarely.

1. Never

2. Occasionally

3. Sometimes

4. Often

5. Always

Question 22: I have frequent awakening at night due to my dream about myself or my family being infected by COVID-19.

1. Never

2. Occasionally

3. Sometimes

4. Often

5. Always

Question 23: I have changes in my eating habits

1. Never

2. Occasionally

3. Sometimes

4. Often

5. Always

Question 24: I have constipation or frequent urination.

1. Never

2. Occasionally

3. Sometimes

4. Often

5. Always

Interpretation of score: A total score; 0-28 is Normal; >28 and ≤ 51 indicates mild to moderate distress; ≥52 indicates severe distress.

There are various volunteer organization / Individual in Nepal who provide psychosocial support for your mental health problem. If you feel you are in distress please seek counseling support. Below is contact of one volunteer organization in Nepal.

Counseling Psychology Nepal

NB: you can contact one of our researcher and psychiatrist
